# Predicted health care profile after transition to adult care in Turner syndrome children—experience of single center

**DOI:** 10.3389/fped.2023.1173419

**Published:** 2023-05-30

**Authors:** Ewa Witkowska-Krawczak, Michał Erazmus, Anna Majcher, Beata Pyrżak, Anna Małgorzata Kucharska

**Affiliations:** Department of Pediatrics and Endocrinology, Medical University of Warsaw, Warsaw, Poland

**Keywords:** turner syndrome, short stature, karyotype, puberty, congenital heart disease, autoimmune thyroiditis, quality of life

## Abstract

**Introduction:**

Turner Syndrome (TS) is caused by the complete or partial loss of one of the X chromosomes in all or some female cell lines. The variable genotypes are responsible for a large phenotypic diversity, nevertheless most studies emphasize a weak correlation between genotype and phenotype. The study aimed to assess the occurrence of defects and diseases depending on the karyotype in patients with TS and correlation with the predicted health care profile after the transition to adulthood.

**Materials and methods:**

45 patients of the Department of Endocrinology and Pediatrics of the Medical University of Warsaw in 1990–2002 were analyzed. Girls were divided into 2 subgroups: “A”, which included 16 patients with the karyotype 45,X, and “B”, which included 29 girls with mosaic karyotypes. Based on the literature data, characteristic phenotypic features and the typical defects or diseases accompanying TS were selected, and the frequency of their occurrence was compared in both subgroups. Accordingly to this data, the predicted medical care profile was determined.

**Results:**

In our study, patients with complete monosomy of the X chromosome had more characteristic phenotypic features. They needed sex hormone replacement therapy more often and started to menstruate spontaneously much less frequently (only 18.18% in monosomy vs. 73.91% in mosaic patients, *p* = 0.006). In patients with monosomy, congenital defects of the circulatory system were found more often (46.67% vs. 30.77%). The diagnosis in patients with mosaic karyotype was more often delayed, therefore the optimal time of growth hormone therapy was shorter. In our study, the X isochromosome determined the higher prevalence of autoimmune thyroiditis (83.33% vs. 12.5%, *p* = 0.049). We didn't find a correlation between the type of karyotype and health care profile after the transition, most of the patients needed more than 2 specialists. Most often, they required: gynecologists, cardiologists, and orthopedics.

**Conclusions:**

After the transition from pediatric to adulthood, patients with TS need multidisciplinary care, but not all need the same kind of assistance. The phenotype and comorbidities determine the profile of patients' health care, however it wasn't directly related to the type of karyotype in our study.

## Introduction

Turner Syndrome (TS) was described in 1938 by Henry Hubert Turner (1). It occurs with a frequency of approximately 1:2.500 live female births. It is estimated that about 100 girls with TS are born annually in Poland (2). The syndrome is caused by the complete or partial loss of one of the X chromosomes in all or some of the female cell lines. Girls with TS are most frequently characterized by short stature, dysmorphic features and gonadal dysgenesis. They suffer more often than the general population from congenital defects of the circulatory system, urinary tract, endocrine disorders, eye and hearing disorders, and autoimmune diseases. There is a large phenotypic diversity. Current knowledge on the mechanisms of phenotypic features in TS is still incomplete. Most studies emphasize a weak correlation between genotype and phenotype (3). However the progress of genetic and molecular diagnostics allows for the identification of an increasing number of genes within the X chromosome responsible for the occurrence of individual symptoms (4). The kind of disorders associated with TS determines the kind of specialistic medical care for the whole life.

The study aimed to assess the occurrence of defects and diseases depending on the karyotype in young patients with TS and its correlation with predicted future health care profile after the transition to adulthood.

## Materials and methods

45 patients who were in the childhood under the care of the Department of Endocrinology and Pediatrics in 1990–2020, were qualified for the study. The inclusion criterion for the study was the diagnosis of TS, based on the cytogenetic examination of at least 20 peripheral blood cells, analyzing metaphases from the standard culture with the formula 550–400 bands. There was no exclusion criteria. Based on the collected medical records, a retrospective analysis was performed.

**Figure 1:**
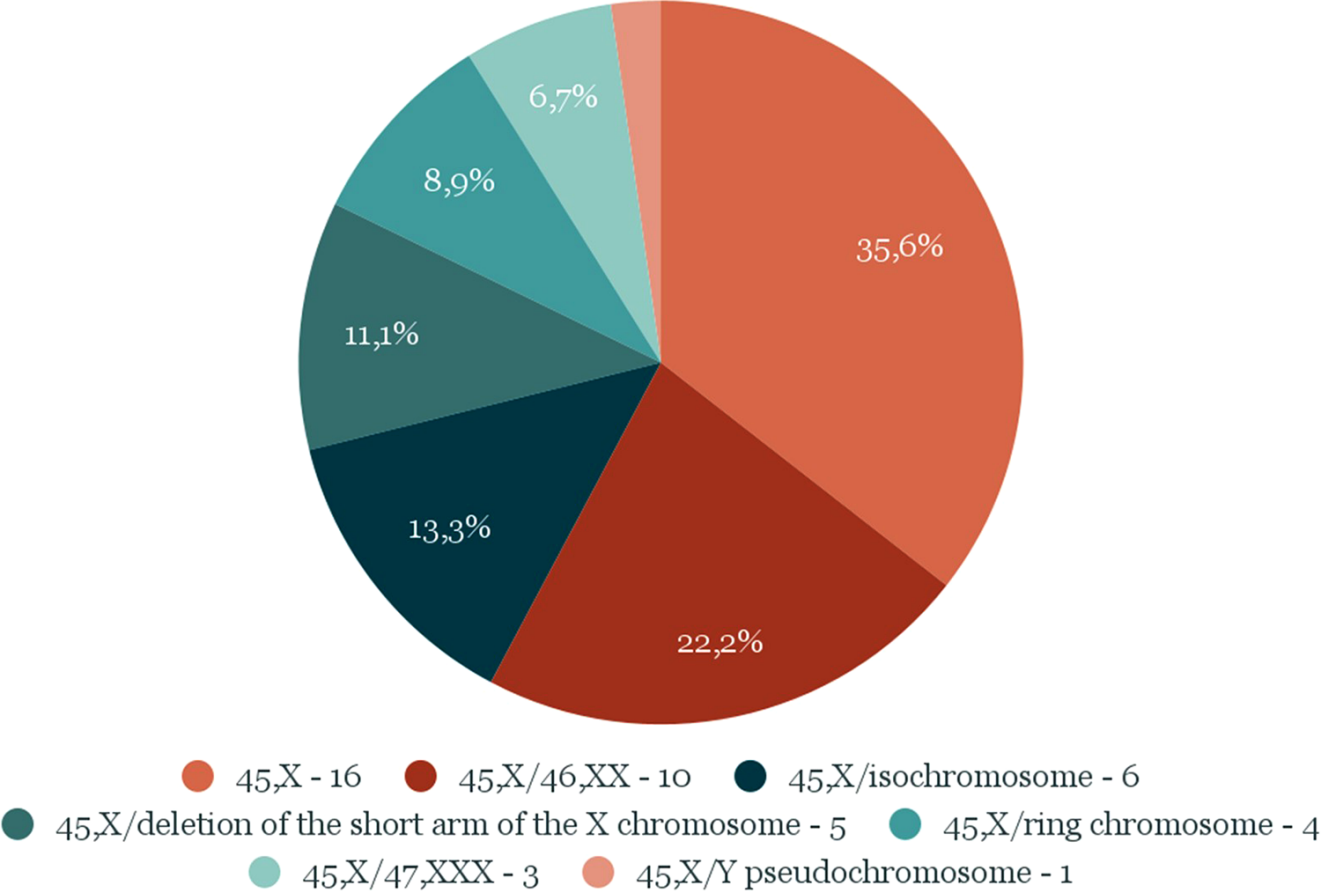
Number and percentage distribution of types of karyotypes in the study group.

The study group was divided into 2 subgroups: “A”, which included 16 patients with the karyotype 45, X, and “B”, which included 29 girls with mosaic karyotypes, regardless of their type. The distribution of particular types of karyotypes is presented in Figure 1.

Based on the “Clinical practice guidelines for the care of girls and women with Turner syndrome” (5), the frequency of TS diagnosis in both subgroups was analyzed according to age:

(1) <6 years of age. Optimal for the early initiation of treatment with recombinant human growth hormone (rhGH),
(2) 6–12 years of age—delayed implementation of rhGH treatment, optimal pubertal monitoring,
(3) > 12 years of age—suboptimal medical care, too late initiation of treatment (5).

**Figure 2:**
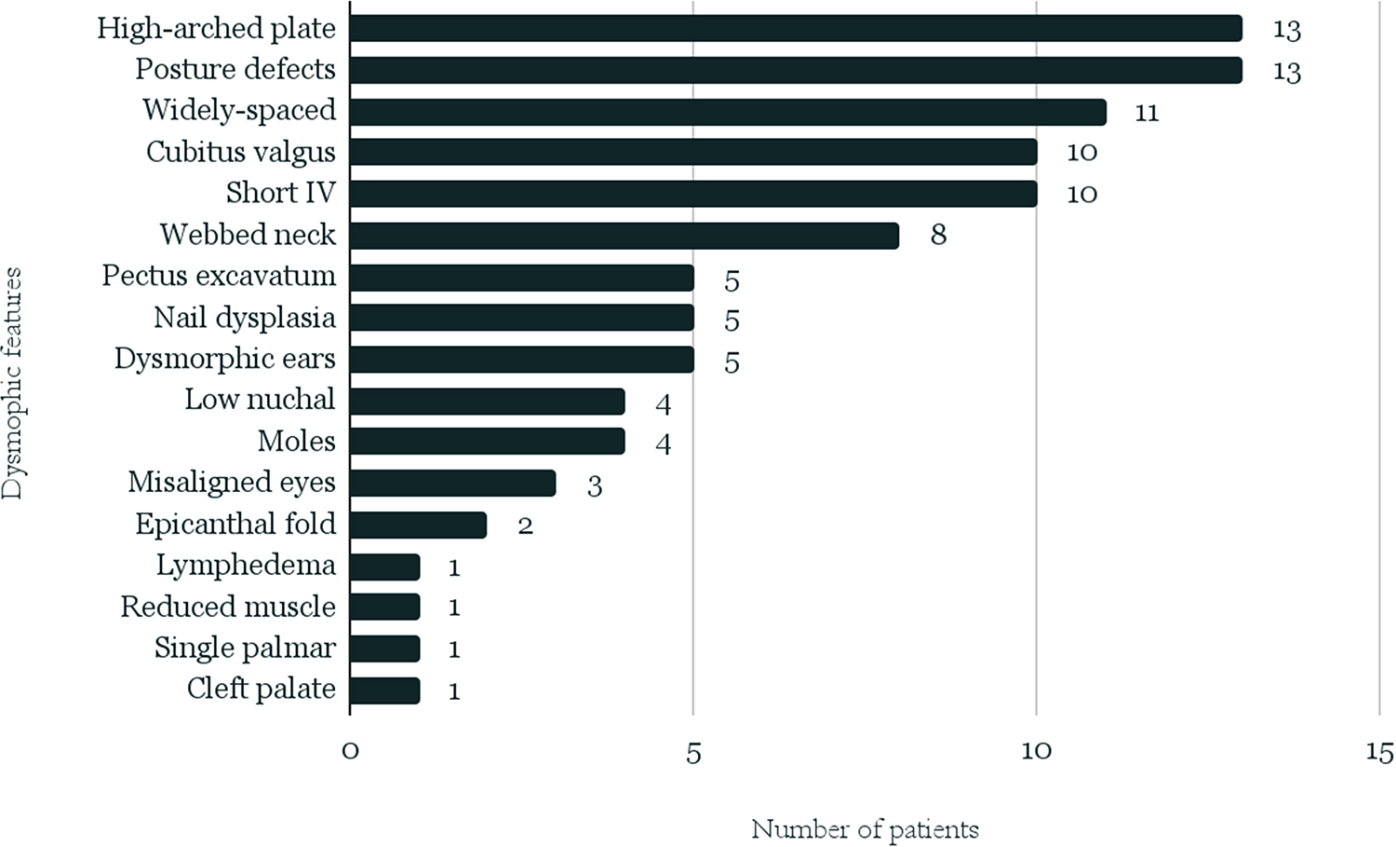
Distribution of the analyzed phenotypic traits among patients from the whole study group.

Characteristic phenotypic features and the typical defects or diseases most often accompanying TS were selected (Table 1, Figure 2), and the frequency of their occurrence was analyzed in subgroups dependently on karyotype.

**Table 1:** Distribution of the analyzed features among patients from the whole study group and “A” and “B” subgroups.

	Group “A”—45,X (*N* = 16)	Group “B”—mosaic chromosomes (*N* = 29)	Whole group (*N* = 45)	No data
Age of diagnosis				
<6 year of old	4/16 (25%)	4/29 (13,79%)	8/45 (17,78%)	
6–12 year of old	3/16 (18,75%)	16/29 (55,17%)	19/45 (42,22%)	
>12 year of old	9/16 (56,25%)	9/29 (31,03%)	18/45 (40%)	
**Short stature**	14/14 (100%)	24/26 (92,31%)	38/40 (95%)	5/45 (“A”—2/16; “B”—3/29)
**Final growth**				11/45 (“A”—4/16; “B”—7/29)
<3rd percentile	5/12 (41,67%)	13/22 (59,09%)	18/34 (52,94%)	
3–10th percentile	5/12 (41,67%)	8/22 (36,36%)	13/34 (38,24%)	
10–25th percentile	2/12 (16,67%)	1/22 (4,55%)	3/34 (8,82%)	
**Phenotypic features**				5/45 (“A”—2/16; “B”—3/29)
≤2	5/14 (35,71%)	15/26 (57,69%)	20/40 (50%)	
>2	9/14 (64,29%)	11/26 (42,31%)	20/40 (50%)	
**Spontaneous onset of estrogen-dependent sexual development (patients >13 year of old)**	2/11 (18,18%)*	17/23 (73,91%)*	19/34 (55,89%)	5/11 (“A”—5/16; “B”—6/29)
**Spontaneous onset of menstruation (patients >15 year of old)**	2/11 (18,18%)*	14/20 (70%)*	16/31 (51,61%)	14/45 (“A”—5/16; “B”—9/29)
**Cardiovascular system defects**	7/15 (46,67%)	8/26 (30,77%)	15/40 (37,5%)	4/45 (“A”—1/16; “B”—3/29)
Bicuspid aortic valve	4/15 (26,67%)	2/26 (7,69%)	6/40 (15%)	
Aortic regurgitation	3/15 (20%)	2/26 (7,69%)	5/40 (12,5%)	
Coarctation of the aorta	2/15 (13,33%)	2/26 (7,69%)	4/40 (10%)	
Patent foramen ovale	1/15 (6,66%)	2/26 (7,69%)	3/40 (7,5%)	
Aortic stenosis	0/15 (0%)	3/26 (11,54%)	3/40 (7,5%)	
Patent ductus arteriosus	1/15 (6,66%)	1/16 (3,85%)	2/40 (5%)	
Atrial septal defect	2/15 (13,33%)	0/26 (0%)	2/40 (5%)	
**Autoimmune thyroiditis**	3/16 (18,75%)	6/29 (20,69%)	9/45 (20%)	
**Urinary system defects**	2/15 (13,33%)	4/27 (14,81%)	6/42 (14,29%)	3/45 (“A”—1/16; “B”—2/29)
**Hearing disorders**	2/14 (14,29%)	10/23 (43,48%)	12/37 (32,43%)	8/45 (“A”—2/16; “B”—6/29)
**Vision disorders**	1/14 (7,14%)	6/22 (27,27%)	7/36 (19,44%)	9/45 (“A”—2/16; “B”—7/29)
**Celiac disease**	0/14 (0%)	2/25 (8%)	2/39 (5,13%)	6/45 (“A”—2/16; “B”—4/29)

*Statistically significant data (*p*-value <0.05).

Patients older than 13-year-old (upper age limit of puberty initiation) were selected and analyzed for possible onset of puberty, (Tanner score, serum estradiol and LH, FSH concentrations, ovarian size in ultrasound). Also, patients older than 15-year-old (upper age limit of menarche) were verified for spontaneous onset of menstruation.

All the above-described data were statistically processed using the chi-Square Calculator program.

On the basis of the above-developed data, a projected profile of specialist care after the age of 18 was created for each patient, depending on the health burdens.

## Result

### Age of diagnosis

The age of TS diagnosis in the study group ranged from the prenatal period to 17 years old. The average age of the karyotype testing was 10.16 years. Among patients with X chromosome monosomy, it was 10.56 years; among those with mosaic karyotypes, it was 9.93 years. We found no statistically significant difference between the mean age of diagnosis throughout the 3 decades of our observation.

In group “A” in 4/16 (25%) patients the diagnosis of TS was placed <6 years of age, in 3/16 (18.75%) patients between 6 and 12 years of age, in 9/16 (56.25%) the diagnosis was too late, >12 years of age. In group “B”, the karyotype was determined in 4/29 (13.79%) patients <6 years of age, in 16/29 (55.17%) between 6 and 12 years of age, in 18/29 (40%) > 12 years of age (Table 1). There was no statistically significant difference in the age of diagnosis of the syndrome in the compared groups (*p*-value >0.05).

### Growth

All girls with a karyotype 45, X had body height below 3 percentile for calendar age and received recombinant human growth hormone (rhGH) therapy. Among patients with mosaic karyotypes, height deficiency was diagnosed in 24/26 (92.31%), and all of them were treated with rhGH in the Program for Treatment of Short Children with TS. In 2/26 patients (7.69%), height was within the normal range for age (Table 1) and they did not require the rhGH treatment. Unfortunately, in the 5 oldest patients—2 from group “A” and 3 from group “B”, retrospective data on body height have not been available.

In 34 patients—12 from group “A” and 22 from group “B”, the final body height after the end of growth was also determined. Among patients with X chromosome monosomy, body height <3rd percentile was found in 5 (41.67%), 5 (41.67%) had height in the 3rd–10th percentile channel, and 2 (16.67%) in 10th–25th percentile channel. Among patients with mosaic karyotype, 13 (59.09%) completed growth <3 percentile, 8 (36.36%) in the 3rd–10th percentile channel, 1 (4.55%) > 10 percentile (Table 1). The higher described differences were not statistically significant (*p*-value > 0.05).

### Phenotypic features

Data of characteristic phenotypic features could be analyzed in 40 patients—14 from the “A” group and 26 from the “B” group. The list of the traits and their distribution in the entire study group are presented in Figure 2.

Depending on the number of phenotypic features, the following patients from both subgroups were compared:

(1) with a maximum 2 features—as an incomplete phenotypic picture of TS, which may occur incidentally and not be associated with the diagnosis, and
(2) having more than 2 characteristic features—as a phenotypic image clearly indicating the diagnosis of TS. In group “A” 2 or less of the features mentioned above were found in 5 (35,71%) patients, and in another 9 (64,29%), there were more phenotypic features. In group B, at least 2 traits were found in 15 (57,69%) girls, and the remaining 11 (42,31%) had more than 2 features (Table 1). No statistically significant difference was found between the type of karyotype and the number of phenotypic features (*p*-value > 0.05). It was also not seen that one of the features occurs significantly more often in combination with any of the types of karyotypes.

### Puberty

Data concerning puberty was analyzed in 11 patients in group “A” and 23 in group “B” at the age of 13 years and older. As expected, pubarche was observed in 100% of them. Spontaneous onset of estrogen-dependent sexual development, assessed on the basis of mammary gland development, ovarian enlargement and increase in serum estrogen concentration, was found in 2 patients from the “A” group (18.18%) and 17 patients from the “B” group (73.91%), which was a statistically significant difference (*p*-value 0.0022*).

The number of patients at the age older than 15 years, in whom the spontaneous onset of puberty was analyzed, was 11 in group “A” and 20 in group “B”. Menarche occurred in 2 girls with karyotype 45, X (18.18%) and 14 with mosaic karyotypes (70%)—this difference is also statistically significant (*p*-value 0.00574*).

All patients without spontaneous onset of puberty received estrogen replacement. Due to the preferences of the patients, 100% of them used the oral form of therapy. It was calculated that the average age of introduction therapy was 13.46-year-old.

### Cardiovascular defects

Data on cardiovascular defects was gathered in 40 patients, including 15 in group “A” and 26 in group “B”. The 3 most frequently detected defects were: bicuspid aortic valve (15% of patients), aortic regurgitation (12.5%), and aortic coarctation or subcoarctation (10%). Among patients with monosomy of the X chromosome, cardiovascular defects were found in 7/15 (46.67%), while among those with mosaicism in 8/26 (30.77%)—a statistically insignificant difference (*p*-value > 0.05). The types of defects and the percentage of patients with them in each group are presented in Table 1. There was no significant predominance of any of the defects in any of the subgroups.

### Autoimmune thyroiditis

Autoimmune thyroiditis was diagnosed in 3/16 (18.75%) girls from group “A” and 6/29 (20.69%) girls from group “B”. Due to reports in the literature regarding the correlation of the occurrence of autoimmune thyroiditis with the isochromosome (15–18), this aspect was also checked. In the entire study group, autoimmune thyroiditis was found in 9/45 patients (20%), while in the group of girls with isochromosome in 5/6 (83.33%). A statistically significant difference was found between the number of patients with autoimmune thyroiditis in the isochromosome group (5/6) and among girls with all other karyotypes (4/39)—*p*-value 0.000031.

### Other defects and accompanying diseases

Both subgroups of patients were also analyzed for the prevalence of urinary tract defects, vision and hearing disorders, celiac disease. The frequency of the above-described diseases in both studied subgroups is shown in Table 1. The patients were diagnosed with: horseshoe kidney, double or dilated calyx-pelvic system, upper calyx syndrome, chronic or recurrent exudative otitis media, hearing loss, myopia, astigmatism, monergism, and convergent strabismus. None of the diagnoses occurred significantly more often in patients with monosomy X chromosome than in patients with mosaic karyotypes.

### Predicted specialist care after the age of 18

Based on the data described above and Clinical practice guidelines for the care of girls and women with Turner syndrome (5), a predictive profile of specialist care after the age of 18 was created for patients in the study group, which is presented in Table 2.

**Table 2:** A predictive profile of specialist care in the study group after the transition to adult care.

Specialist	Number of patients	Health aspects	Recommendations (5)
Primary care	All patients	Special prevention	TSH, fT4, HbA1c, AST, ALT, GGT, ALP, clinical evaluation for scoliosis every year
Gynecologist	All patients	Prevention as in a healthy population	
		Persistent ovarian function	Observation for premature ovarian failure
			Consider oocyte cryopreservation
		Hypogonadism	Hormone Replacement Therapy
			Consider assisted reproductive therapy
Cardiologist	All patients	Special prevention	Transthoracic echocardiography (TTE) or cardiac magnetic resonance scan (CMR) every 10 years
		Pregnancy	TTE and CT/CMR 2 years before planned pregnancy
			TTE about 20 weeks of gestation
		Cardiovascular system defects—control and treatment	
Otolaryngologist	All patients	Special prevention	Audiometric evaluation every 5 years
		Hearing disorders—control and treatment	
Orthopaedist and rehabilitation specialist	15	Posture defects, valgus of the limbs—control and treatment	
Endocrinologist	9	Autoimmune thyroiditis—control and treatment	
Ophthalmologist	7	Vision disorders—control and treatment	
Dermatologist	4	Multiple moles—control	
Nephrologist	6	Urinary system diseases—control and treatment	
Gastroenterologist	2	Celiac disease—control and treatment	

## Discussion

Due to such a diverse genotypic and phenotypic picture of TS, numerous studies are being conducted to clarify the relationship between the type of genotype and the clinical picture of the disease, however there are not many studies trying to find key factors determining the type and quality of health care profile after the transition to adulthood.

During the pediatric period, the care of TS patients is usually well coordinated by the pediatric endocrinologists, because of rhGH treatment and puberty induction. However after the transition to adult care, medical management becomes decentralized and divided among several specialists. An efficient transition process and one center coordinating the entire treatment should improve the quality of life (QoL) of TS patients. Many studies and recommendations emphasize the significantly lower QoL of adult patients with TS, so efforts should be made to improve this factor on many levels, starting primarily with the quality of medical care and effective transition (4). The QoL is dependent also on a proper education about the TS associated problems, additionally strong emotional support for these patients and constant psychological care, especially in adulthood should not be underestimated.

One of the most significant factors affecting the QoL of patients with TS is short stature, which is also the most frequent reason for the start of diagnostics. Indeed, short stature is the most common feature of girls with TS with classical monosomy as well as mosaic karyotypes (8). The earlier TS diagnosis allows for early rhGH treatment, however our study reveals that many patients with TS remain short adults. In our study group the final height position below 3rd percentile was present in 41,67% of patients with 45, X0 karyotype and 59,09% of patients with mosaic karyotypes.

Our study confirms the previously published observations, that the characteristic dysmorphic features of TS are more discreetly expressed in patients with a mosaic karyotype (6). This fact explains a general tendency towards delayed diagnosis in this group. In our study the average age of diagnosis of TS was 10,16 years, and there was no significant difference between the groups of patients with particular karyotypes (10,56 in patients with monosomy vs. 9,93 in patients with mosaicism, *p* > 0,05). A study population bias could explain this paradoxical observation, because mosaicism was presented by our patient almost 2 times more frequently than monosomy, discordantly with known literature data, where the prevalence of monosomy is estimated at 50%. Also, surprisingly, the mean age of diagnosis was much older than the average in many studies conducted in Western countries (19, 20). It indicates that the most frequent reason for the diagnostics in TS girls was short stature. It was the main characteristic feature that became more evident in school children.

Our study highlights the importance of karyotyping in all girls with short stature (21). The gold standard for karyotype evaluation is the examination of peripheral blood leukocytes. According to the guidelines, such an analysis should contain at least 20 cells because this methodology identifies at least 10% mosaic karyotypes with 95% sensitivity (9–11). It means that some patients with a mosaic karyotype can be overlooked with the standard diagnostic course, hence may never receive proper growth-promoting therapy or may never be diagnosed with comorbidities and then not receive appropriate specialist care, also after the transition to adulthood (12–14).

The results of our study revealed that the prevalence of comorbidities and systemic defects is similar in girls with a mosaic karyotype and classical monosomy, which was also reported by other authors (7, 8). It seems justified to highlight in the studies that the weak expression and sometimes even lack of characteristic dysmorphic features (so common in patients with a mosaic karyotype) cannot diminish vigilance and cause less active monitoring of comorbidities. It should be highlighted in the guidelines for pediatricians and physicians when the transition process is planned.

In our database, less than 55% of girls required hormonal induction of menstruation, while the up-to-date literature mentioned as much as 80%–90% (18). It probably was caused mainly due to the relatively high percentage of patients with mosaic karyotypes in our study group. In our study, there was an advanced average age of initiating estrogen replacement, followed by a delayed age of reaching the full sex hormone replacement. In the majority of patients, it was dependent on the desire to prolong the growth-promoting treatment delayed by the late TS diagnosis. It seems fully justified to ask whether the transition to adulthood care of TS patients should be postponed in such patients over the age of 18 years until they complete puberty. A separate problem noticed in our research is the preference of TS young girls for oral formulas of sex hormone replacement therapy. The main reason reported by girls and their parents was usually the fear of stigmatization in the peer environment when they use plasters. However, transdermal systems are recommended, therefore it seems important to encourage patients to use a more effective and safe method, both—during pediatric care and after the transition to adulthood (22).

A separate factor worsening QoL is gonadal dysgenesis and infertility. Oocyte cryopreservation is recommended for teenagers with TS from the age of 12, who have functioning ovaries. However, more and more studies emphasize the need to educate parents of younger girls with TS and the possible earlier decision about oocyte cryopreservation or ovarian tissue cryopreservation even from the age of 6, as premature ovarian failure generally occurs early in life in young women with TS, rapidly after the process of transition from pediatric care (5, 23, 24). Adult patients with TS will require a slightly different and more flexible approach during gynecological care. Medical staff and parents should start discuss this topic with adolescent girls while providing them with appropriate psychological support.

It should be remembered that in case of pregnancy, during obstetric care, patients with TS require careful cardiological care, frequent echocardiographic monitoring, and strict blood pressure control. Education on greater cardiovascular risk and the principles of a healthy lifestyle should be implemented from early childhood and then continued in internist care in adulthood.

The data available in the literature suggest that specific TS comorbidities are much more common among patients with some variants of mosaic karyotypes. It is well known i.e., that patients with TS suffer from autoimmune diseases more often than the rest of the TS population. In this group, autoimmune thyroiditis (AT) is more frequent, which is mentioned in many studies (25). This applies, for example, to the correlation of AT with the X isochromosome (15–18). Our analysis also confirmed this strong association—the X isochromosome determined the higher prevalence of AT (83.33% vs. 12.5% in monosomy and other karyotypes, *p* = 0.049).

It is essential to screen the presence of other autoimmune diseases in TS children, i.e., celiac disease. The clinical picture of celiac disease often takes atypical forms that can overlap TS symptoms, like menstrual disorders or infertility. The disease may develop over the years and could not be diagnosed until adulthood. Therefore, it is justified to emphasize the importance of active screening. It is suggested to consider to check periodically the level of antibodies against tissue transglutaminase also after the transition in girls with TS, especially when suspicious symptoms or signs are present (5).

In childhood and adolescence, over 90% of TS patients require the treatment with rhGH, which is why the care of these girls is usually coordinated by an endocrinologist. In adulthood, however, the coordinating doctor may change. Depending on the local medical care organization, screening tests for diseases connected with TS can be provided by the general practitioner (GP). According to our country, the GP can monitor TSH, fT4 level and antithyroid antibodies, glucose level, blood pressure, BMI. When some abnormalities are detected, the patients could be referred to an adequate specialist. In our opinion, sometimes too much multidisciplinary care can lead to patient overload and reduced QoL, and therefore in some cases “less could be more”.

In our study, we tried to find the key factors predicting the type and quality of health care profile after the transition to adulthood in patients with TS. We showed health problems affecting TS patients. Some require systematic control, while others require active screening, as they may appear in adult life. This highlights the importance of coordinated medical care after the process of transition. However, we didn't find a strong correlation between the type of karyotype and health care profile. Most of the patients needed more than 3 specialists, and mainly they required: gynecological, cardiological, orthopedics, and otolaryngological care.

## Conclusions

An effective transition process can improve the quality of health care and QoL of TS patients. The majority of TS women need multidisciplinary medical care, which generally becomes more decentralized and divided among several specialists after the transition. The phenotype and comorbidities determine the profile of patients' health care, however it wasn't directly related to the type of karyotype. Accordingly to the most common health problem, main cooperation is needed between gynecologists, cardiologists, and orthopedics, as well as significant psychological support which is essential to prepare properly children and parents to the transition.

Data presented in our research show that patients with complete monosomy of the X chromosome have more characteristic phenotypic features of TS. They also start to menstruate spontaneously much less frequently and therefore need hormone replacement therapy more often. Congenital defects of the circulatory system are also found more often in monosomy. On the contrary, autoimmune thyroiditis was more frequent in patients with mosaic karyotypes. On the other hand, the diagnosis in patients with a mosaic karyotype is more often delayed; therefore, the optimal time of growth-promoting therapy is shortened, and these patients also risk less effective monitoring of comorbidities and a less efficient transition from pediatric care to adulthood. We did not find any other correlation between the type of karyotype and the presence of numerous other defects and diseases associated with TS. This implies the need for the same accurate monitoring and screening in all patients with TS, regardless of karyotype, because the quality of medical care in adult life also depends on it.
